# Expression of DNA-repair proteins and their significance in pancreatic cancer and non-cancerous pancreatic tissues of Sprague–Dawley rats

**DOI:** 10.1186/1477-7819-12-32

**Published:** 2014-02-06

**Authors:** Xing-guo Tan, Zhu-lin Yang, Le-ping Yang, Xiong-ying Miao

**Affiliations:** 1 Department of Hepatobiliary Surgery, The Second Hospital of Yueyang, East Baling Road 263#, Yueyang, Hunan 414000, China; 2 Research Laboratory of Hepatobiliary Diseases, Second Xiangya Hospital, Central South University, Changsha, Hunan 410011, China

**Keywords:** 7,12-dimethylbenzanthracene, Animal model, DNA-repair proteins, Immunohistochemistry, Pancreatic neoplasms, Sprague–Dawley rats, Trichostatin A

## Abstract

**Background:**

To establish a model of pancreatic cancer induced by 7,12-dimethylbenzantracene (DMBA) in Sprague–Dawley (SD) rats, and detect the expression of DNA-repair proteins (MGMT, ERCC_1_, hMSH_2_, and hMLH_1_) and their significance in pancreatic cancer and non-cancerous pancreatic tissues of SD rats.

**Methods:**

DMBA was directly implanted into the parenchyma of rat pancreas (group A and group B), and group B rats were then treated with trichostatin A (TSA). The rats in both groups were executed within 3 to 5 months, and their pancreatic tissues were observed by macrography and under microscopy. Meanwhile, the rats in the control group (group C) were executed at 5 months. Immunohistochemistry was used to assay the expression of MGMT, ERCC_1_, hMSH_2_, and hMLH_1_.

**Results:**

The incidence of pancreatic cancer in group A within 3 to 5 months was 48.7% (18/37), including 1 case of fibrosarcoma. The incidence of pancreatic cancer in group B was 33.3% (12/36), including 1 case of fibrosarcoma. The mean of maximal diameters of tumors in group A was higher than that in group B (*P* <0.05). No pathological changes were found in pancreas of group C and other main organs (except pancreas) of group A and group B. No statistical differences were found among the positive rates of MGMT, ERCC_1_, hMSH_2_, and hMLH_1_ in ductal adenocarcinoma and non-cancerous pancreatic tissues of group A (*P* >0.05). The positive rates of MGMT, ERCC_1_, hMSH_2_, and hMLH_1_ were significantly lower in ductal adenocarcinoma than those in non-cancerous tissues of group B (*P* ≤0.05). All pancreas of group C had positive expression of MGMT, ERCC_1_, hMSH_2_, and hMLH_1_ and two cases of fibrosarcoma showed a negative expression.

**Conclusions:**

DMBA, directly implanted into the parenchyma of pancreas, creates an ideal pancreatic cancer model within a short time. TSA might restrain DNA damage related to the genesis and growth of pancreatic cancer in rats. The DNA-repair proteins, including MGMT, ERCC_1_, hMSH_2_, and hMLH_1_, might play an important role in the genesis of pancreatic cancer induced by DMBA in rats.

## Background

Pancreatic cancer is a solid malignancy characterized by its rapid growth and propensity to invade adjacent organs and metastasize. Worldwide, pancreatic cancer causes approximately 213,000 deaths each year. The 1-year survival rate is around 20% and 5-year survival rate is less than 5% in spite of aggressive therapies [1]. Within the last two decades, research has shown that pancreatic cancer is fundamentally a genetic disease caused by inherited germline and acquired somatic mutations in cancer-associated genes, and more and more investigation of molecular pathogenesis has been used in the diagnosis and treatment of pancreatic cancer. To build useful models studying the pathological molecular mechanisms of pancreatic cancer, Rivera et al. directly implanted dimethylbenzanthracene (DMBA) into the parenchyma of the rat pancreas and found a pancreatic cancer incidence of 39% within 10 months [2]; Bockman et al. reported similar studies [3].

Trichostatin A (TSA) is a histone deacetylase inhibitor with a broad spectrum of epigenetic activities. It can up-regulate the expression of several genes and restrain other genes’ expression, thus intervening in the genesis and development of tumors. *In vivo* or *in vitro* experiments have confirmed that TSA could restrain the genesis of some tumors and control tumor progression by restraining tumor angiogenesis and changing the tumor microenvironment [4]. Some studies have shown that TSA acts as a tumor suppressor in human pancreatic cancer cell lines [5,6].

The DNA mismatch repair (MMR) system is an inbuilt security system that can repair DNA mismatch in human cells, and plays an important role in retaining the integrality and stability of genes. The main MMR genes are hMSH_1-6_, hMLH_1-5_ and others, and the methylation of MMR genes and/or the loss of expression of their proteins plays an important role in malignant tumorigenesis [7-11]. O^6^-methylguanine DNA methyltransferases (MGMT) is a high-performance DNA-repair enzyme, which can protect cells from alkylating agent damage and prevent cell carcinogenesis [11-16]. Excision repair cross-complementing gene 1 (ERCC_1_) is a member of the exonuclease repair enzyme family, and its low expression is always related with elevated cancer incidence while its high expression is always related with resistance to platinum drugs [17-21].

Since no studies have examined the expression levels of DNA-repair proteins (MGMT, ERCC_1_, hMSH_2_, and hMLH_1_) in pancreatic cancer induced by DMBA and non-cancerous pancreatic cancer tissues in rats, little is known about the effects of MGMT, ERCC_1_, hMSH_2_, and hMLH_1_ on rat pancreatic cancer induced by DMBA. In this study, DMBA was directly implanted into the parenchyma of the pancreas of rats to establish a pancreatic cancer model, and TSA injection was given to establish the intervention group. The expression levels of MGMT, ERCC_1_, hMSH_2_, and hMLH_1_ in pancreatic cancer and non-cancerous pancreatic tissues was detected and their effect on the process of inducing cancer by DMBA was assessed.

## Methods

### Animal model

Ninety Sprague–Dawley (SD) rats (no sex limit), weighing between 150 and 200 g were used. These rats were randomly divided into three groups: 40 in the pancreatic cancer model group (group A), 40 in the TSA intervention group (group B), and 10 in the control group (group C). The rats were treated with preoperative fasting for 24 hours (no water ban), and 2% amyl-barbital was injected into the abdomen under anesthesia. The rats’ abdomens and parenchyma were then dissected (1 mm) and DMBA (9 mg) was directly implanted into the parenchyma of the pancreas in groups A and B, followed by suturing. The rats were raised in common conditions after operation, and rats in group B were injected with 1 mL TSA (1 μg/mL) weekly through the abdomen. Except for natural death, the rats were executed randomly in the third month (7 rats in group A and 6 rats in group B), in the fourth month (10 rats in both groups A and B), and in the fifth month (20 rats in both groups A and B) after operation. Rats in group C, which were treated without DMBA implantation and treated in the same condition as group A, were executed in the fifth month after operation.

The design of this study was approved by the medical ethics commitee of the Second Hospital of Yueyang City.

### Macrography and pathological observation

The livers, gallbladder, stomach, intestine, and lung of rats in groups A and B were observed by macrography. After which the whole pancreatic tissues and some tissues from the liver, gallbladder, stomach, intestines, and lungs of rats were put into 4% formaldehyde for 16 to 18 hours. Conventional paraffin-embedded sections were made with these specimens. Finally the sections were dyed by hematoxylin and eosin staining, and observed under microscopy.

### Immunohistochemical staining of EnVision™

Immunostaining was conducted by use of the ready-to-use, peroxidase-based EnVision™ Detection kit (Dako Laboratories, CA, USA) according to the user manual. Four-micrometer-thick sections were cut from routinely paraffin-embedded tissues.

Rabbit anti-rat MGMT, ERCC_1_, hMSH_2_, and hMLH_1_ monoclonal antibodies were obtained from Cell Signaling Technology, Inc. (Danvers, MA, USA). EnVision™ detection kit was from Dako Laboratories, CA, USA. Cytoplasm and (or) cell nuclei containing brown-yellow granules were defined as positive cells. The percentage of positive cells was calculated from 10 random fields. Cases with ≥25% positive cells were considered positive and cases were otherwise considered negative [22,23]. The positive controls were the positive pancreatic cancer biopsies provided by CST (Cell Signaling Technology, Inc.) while the negative controls were prepared by 5% fetal bovine serum substituting the primary antibody.

### Statistical analysis

Data was analyzed by using the statistical package for the Social Sciences Version 13.0 (SPSS 13.0). All the data were analyzed by using χ^2^ test, rank-sum test, and Fisher’s exact test.

## Results

### Macrography

The incidence of pancreatic tumors in groups A and B are shown in Table 1; the incidence of tumors in group A was higher than that in group B (*P* >0.05). Both groups A and B had one case of fibrosarcoma that developed liver metastasis and epiploon metastasis. The distribution of diameter of tumor mass in group A was 0.5–1.0 cm (7 cases), 1.0–2.0 cm (10 cases), and >2.0 cm (1 case); and the distribution of diameter of tumor mass in group B was 0.5–1.0 cm (9 cases), 1.0–2.0 cm (2 cases), and >2.0 cm (1 case). The mean of maximal diameter of tumors in group A was higher than that in group B (*P* <0.05). No pathological changes were found by macrography in pancreas of group C and other main organs (except pancreas) of groups A and B.

**Table 1:** **Incidences of pancreatic tumors in groups A and B** (**case number ratio (%))**

**Group**	**n**	**3**^**rd **^**month**	**4**^**th **^**month**	**5**^**th **^**month**	**Total cases**
A	37	2/7 (28.6)	4/10 (40.0)	12/20 (60.0)	18/37 (48.7)
B	36	1/6 (16.7)	3/10 (30.0)	8/20 (40.0)	12/36 (33.3)

### Pathological observation

**Figure 1:**
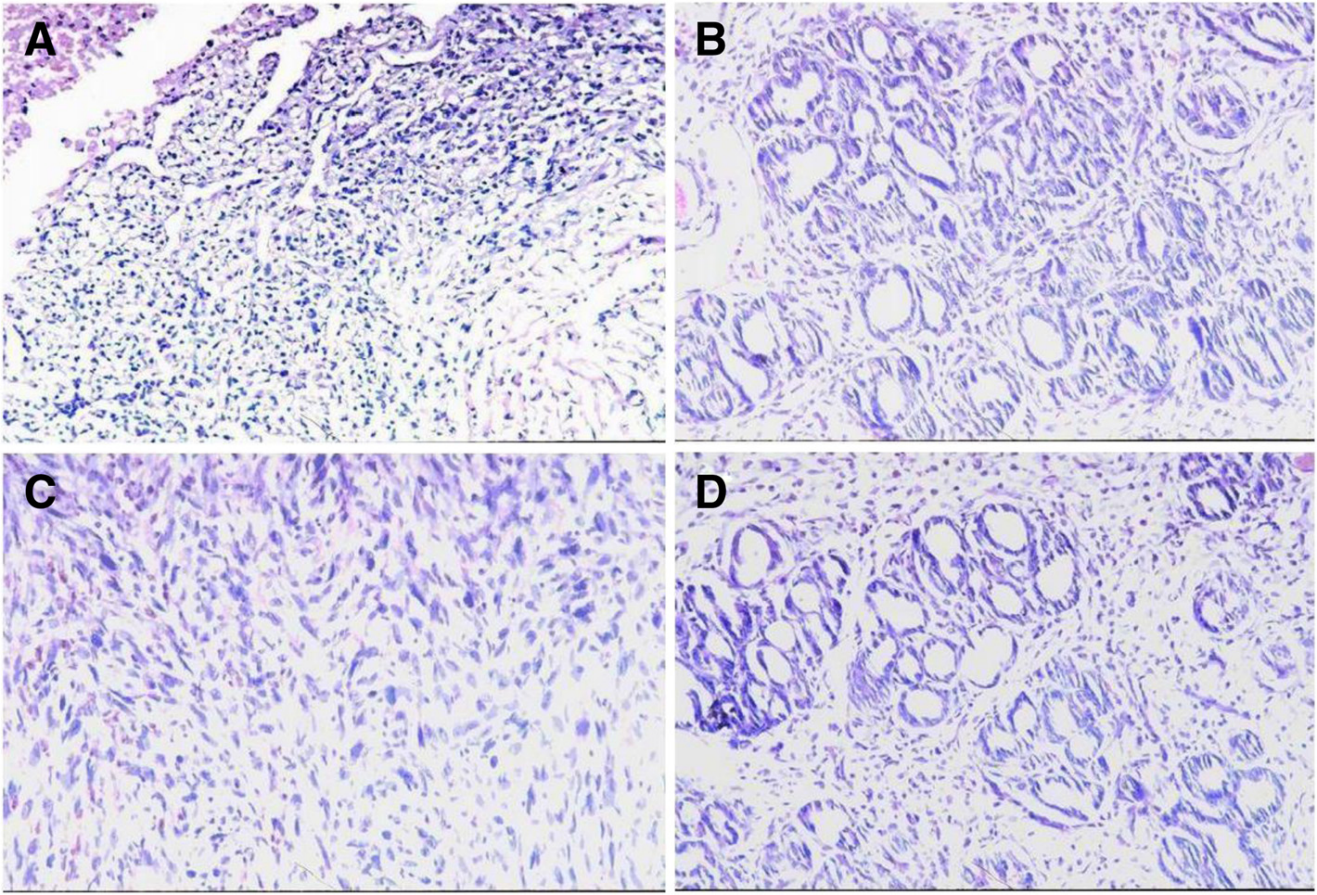
**Pathological observation of rat pancreatic lesions induced by DMBA.** Hematoxylin-eosin stain, original magnification × 200. **(A)** Poorly-differentiated ductal adenocarcinoma (group A); **(B)** Well-differentiated ductal adenocarcinoma (group B); **(C)** Fibrosarcoma (group B); **(D)** Severe atypical hyperplasia in ductal epithelia (group A).

Pathological results of pancreatic tumors in groups A and B are shown in Table 2 and Figure 1A. Both non-cancerous pancreatic tissues and peritumoral pancreatic tissues in groups A and B showed hyperplasia to atypical-hyperplasia. Non-cancerous pancreatic tissues in group A which showed mild atypical-hyperplasia were found in 5 cases (26.3%) and moderately to severely atypical-hyperplasia in 10 cases (52.6%). The same tissues were found in group B in 10 cases (41.6%) and 8 cases (33.3%), respectively; therefore, no statistical differences were found in the two groups (*P* >0.05). No pathological changes were found by microscopy in pancreas of group C and other main organs (except pancreas) of groups A and B.

**Table 2:** Pathological types of pancreatic tumors in groups A and B (case number)

**Group**	**n**	**Fibro-sarcoma**	**Pancreatic ductal adenocarcinoma**		
			**Well-differentiated**	**Moderately-differentiated**	**Poorly-differentiated**
A	18	1	6	7	4
B	12	1	6	4	1

### Expression of MGMT, ERCC_1_, hMSH_2_, and hMLH_1_ in pancreatic ductal adenocarcinoma and non-cancerous pancreatic tissues

**Figure 2:**
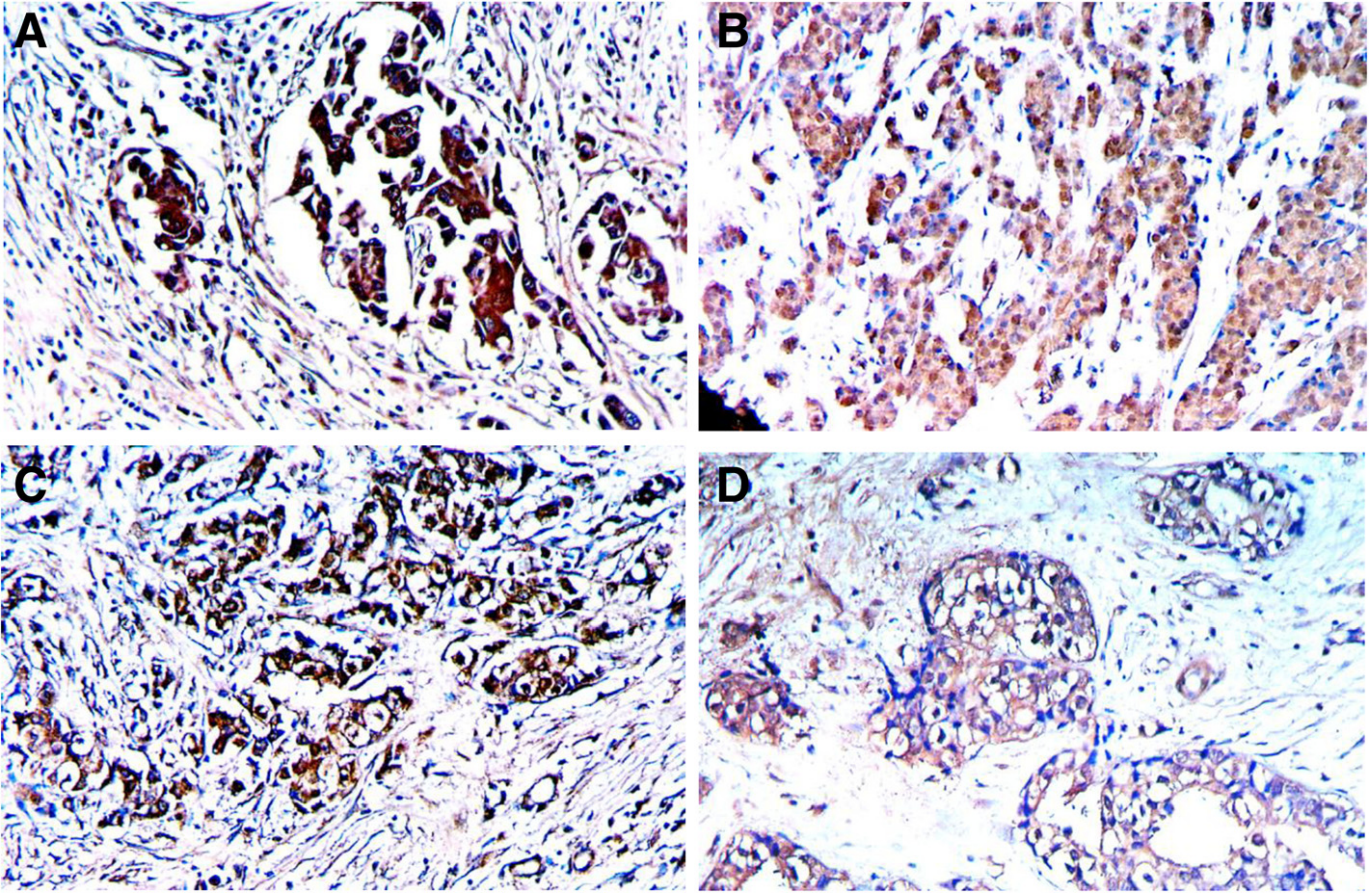
**DNA repair proteins expression in the ductal carcinoma of rat pancreas.** EnVision immunohistochemistry, original magnification × 200 and positive reaction was mainly localized in the cytoplasm and/or nucleus. **(A)** MGMT positive expression in well-differentiated adenocarcinoma; **(B)** ERCC_1_ positive expression in moderately-differentiated adenocarcinoma; **(C)** hMSH_2_ positive expression in moderately-differentiated adenocarcinoma; **(D)** hMLH_1_ positive expression in well-differentiated adenocarcinoma.

The positive rates of MGMT, ERCC_1_, hMSH_2_, and hMLH_1_ (Figure 2) were significantly lower in ductal adenocarcinoma than those in non-cancerous pancreatic tissues in group A + group B (*P* <0.01 or *P* <0.05). No statistical differences were found among the positive rates of MGMT, ERCC_1_, hMSH_2_, and hMLH_1_ in ductal adenocarcinoma and non-cancerous pancreatic tissues of group A (*P* >0.05). The positive rates of MGMT, ERCC_1_, hMSH_2_, and hMLH_1_ were significantly lower in ductal adenocarcinoma than those in non-cancerous tissues of group B (*P* <0.05). The ductal epithelium of non-cancerous pancreas which had negative expression of MGMT, ERCC_1_, hMSH_2_, and hMLH_1_ in groups A and B all showed moderately or severe atypical-hyperplasia. The fibrosarcoma had negative expression of MGMT, ERCC_1_, hMSH_2_, and hMLH_1_, while pancreas of group C had positive expression of MGMT, ERCC_1_, hMSH_2_, and hMLH_1_ (Table 3). Expression of MGMT, ERCC_1_, hMSH_2_, and hMLH_1_ had no obvious correlation with the size of tumor mass and differentiation degree of ductal adenocarcinoma (*P* >0.05).

**Table 3:** **Expression of MGMT**, **ERCC**_**1**_, **hMSH**_**2**_, **and hMLH**_**1 **_**in ductal adenocarcinoma and non**-**cancerous pancreatic tissues (%)**

**Group**	**n**	**MGMT**	**ERCC**_**1**_	**hMSH**_**2**_	**hMLH**_**1**_
Pancreatic ductal adenocarcinoma					
A + B	28	15 (53.6)	13 (46.2)	14 (50.0)	12 (42.9)
A	17	9 (52.9)	8 (47.1)	9 (52.9)	8 (47.1)
B	11	6 (54.6)	5 (45.5)	5 (45.5)	4 (36.4)
Non-cancerous pancreatic tissues					
A + B	43	35 (81.4)	33 (76.7)	32 (74.4)	30 (70.0)
A	19	14 (73.7)	14 (73.7)	12 (63.2)	11 (57.9)
B	24	21 (87.5)	19 (79.2)	19 (79.2)	19 (79.2)

Pancreatic ductal adenocarcinoma compared with non-cancerous pancreatic tissues in group A + B: *χ*^2^_MGMT_ = 6.30, *P* <0.05; *χ*^2^_ERCC1_ = 6.83, *P* <0.01; *χ*^2^_hMSH2_ = 4.43, *P* <0.05; *χ*^2^_hMLH1_ = 5.08, *P* <0.05.

Pancreatic ductal adenocarcinoma compared with non-cancerous pancreatic tissues in group A: *P*_MGMT_ = 0.157, *P*_ERCC1_ = 0.088, *P*_hMSH2_ = 0.103, *P*_hMLH1_ = 0.452.

Pancreatic ductal adenocarcinoma compared with non-cancerous pancreatic tissues in group B: *P*_MGMT_ = 0.042, *P*_ERCC1_ = 0.050, *P*_hMSH2_ = 0.050, *P*_hMLH1_ = 0.018.

## Discussion

Establishing of a pancreatic cancer model can be achieved through three kinds of methods [24-27]: 1) exposing canine animal to carcinogen, 2) activating the oncogenes of transgenic mice, and 3) transplanting the xenogenic pancreatic cancer tissues to athymic mouse. Rivera et al. directly implanted DMBA into the parenchyma of rat pancreas to establish a pancreatic cancer model of rats and the incidence of cancer of SD rats within 10 months was 39% [2]. Since then, a series of mouse and rat pancreatic cancer models using DMBA have been established [28-32]. TSA can increase intracellular histone levels and up-regulate the expression of several genes. Some experiments have confirmed that TSA can restrain the genesis of some tumors by restraining angiogenesis, inhibiting proliferative activity, and promoting apoptosis of tumor cells [33-37]. After we directly implanted a major dose of DMBA (9 mg) into the pancreas parenchyma of SD rats, the incidence of cancer in group A within 3 to 5 months was 48.7%, and that in group B was 33.3%; their pathological types were the same as those of human pancreatic ductal adenocarcinoma, except for two cases of fibrosarcoma. The incidence of cancer in group A was higher than that in group B, but the difference had no statistical significance (*P* >0.05). The mean of maximal diameter of tumors in group A was higher than that in group B (*P* <0.05). Our SD rat model of pancreatic cancer had some merits: 1) the period of tumor formation was short and the incidence of cancer was high; 2) the pathological type was mainly the same as human pancreatic ductal adenocarcinoma; 3) no pathological changes were found in main organs (except pancreas); 4) the inhibitive effect on carcinogenesis and growth of TSA was obvious; and 5) the cost was low.

MGMT is a high-performance DNA-repair enzyme that can protect cells from alkylating agent damage and can prevent cell carcinogenesis and death. The *MGMT* gene is located in 10q^26^ and encodes 207 amino acids’ proteins [7-11]. Normal cells all have MGMT expression, while some malignant tumors will lose MGMT expression which will induce the damage of DNA repair and the carcinogenesis of cells [7-11,38,39]. ERCC_1_ is a member of the exonuclease repair enzyme family and its low expression is always related to elevated cancer incidence, while its high expression is always related to resistance to platinum drugs [12-16]. Recent studies have confirmed that ERCC_1_ is the key enzyme of the DNA repair induced by cisplatin and it has been shown that ERCC_1_ expression of some malignant tumors played an important role in guiding chemotherapy [17-21,37]. The *hMSH*_*2*_ gene is located in 2P^16^ and is the first separated MMR. It can repair DNA mismatch and retain the integrality and stability of genes. Many recent papers have reported that the loss of hMSH_2_ protein expression was crucial to the genesis and progression of malignant tumors [7-11,40-42]. hMLH_1_ is also a type of MMR which can also inhibit carcinogenesis by repairing DNA mismatching. Mutation of the *hMLH*_*1*_ gene will induce the genesis of many malignant tumors [7-11,41].

## Conclusions

Our data have shown that the positive rates of MGMT, ERCC_1_, hMSH_2_, and hMLH_1_ were significantly lower in pancreatic ductal adenocarcinoma than in non-cancerous pancreatic tissues of rats, and the ductal epithelia of non-cancerous pancreas which had negative expression of MGMT, ERCC_1_, hMSH_2_, and hMLH_1_ all shown atypical-hyperplasia. The results show that there was loss expression of MGMT, ERCC_1_, hMSH_2_, and hMLH_1_ in the course of genesis of pancreatic cancer induced by DMBA in rats, which might be the mechanism of carcinogenesis by DMBA. Therefore, testing the expression of MGMT, ERCC_1_, hMSH_2_, and hMLH_1_ in pancreatic cancer might play an important role in guiding the treatment of human pancreatic cancer.

## Abbreviations

DMBA: Dimethylbenzanthracene; ERCC1: Excision repair cross-complementing gene 1; MGMT: O^6^-methylguanine DNA methyltransferases; MMR: Mismatch repair; SD: Sprague–Dawley; TSA: Trichostatin A.

## Competing interests

The authors report no competing interests. No benefits in any form have been received or will be received from a commercial party related directly or indirectly to the subject of this article.

## Authors' contributions

TXG did most of the experiments and data acquisition, TXG and YZ participated in the design of experiments, interpretation of data, and writing of the manuscript. LY and XYM participated the experiments and writing. All authors read and approved the final manuscript.
